# Retrospective analysis of heroin detoxification with buprenorphine in a psychiatric hospital in Japan

**DOI:** 10.1002/npr2.12147

**Published:** 2020-10-27

**Authors:** Tatsushi Nagano, Sohei Kimoto, Katsuro Aso, Takashi Komori, Yasunari Yamaguchi, Kazuya Okamura, Noriya Yamamoto, Toshifumi Kishimoto

**Affiliations:** ^1^ Department of Psychiatry Fukko‐kai Tarumi Hospital Kobe Japan; ^2^ Department of Psychiatry Nara Medical University School of Medicine Kashihara Japan

**Keywords:** buprenorphine, Clinical Opiate Withdrawal Scale, detoxification, heroin, opioid use disorder

## Abstract

**Aim:**

We assessed the efficacy of buprenorphine replacement taper therapy in a psychiatric hospital in Japan.

**Methods:**

Based on the medical records, a retrospective analysis was performed to evaluate the outcomes of buprenorphine replacement taper therapy in 106 subjects with heroin dependence.

**Results:**

We found that replacement and taper therapy with buprenorphine could significantly reduce withdrawal symptoms during detoxification. In addition, the completion rate of detoxification was significantly improved and the length of hospital stay was significantly reduced relative to those who received conventional treatment without buprenorphine. However, the readmission rate increased after the introduction of detoxication therapy with buprenorphine.

**Conclusion:**

The present findings suggest not only the efficacy and safety of buprenorphine replacement and taper therapy, but also the requirement for maintenance therapy for individuals with heroin dependence.

## Graphical abstract

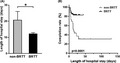

## 1 INTRODUCTION

Heroin is a highly addictive drug produced from morphine, which is a principal component of opium. In addition to its powerful euphoria, heroin induces both psychological and physical dependence, and thus, it is one of the most commonly used illicit drugs worldwide. In the United States, since the 1990s, the widespread use of opioids for noncancer pain has led to a vicious cycle of abuse and resale of these drugs, as well as the abuse of heroin, which can be obtained cheaply, leading to an opioid epidemic^1^ and an opioid overdose crisis.^2^ A recent report revealed that the prevalence of heroin use increased from 0.17% in 2002 to 0.32% in 2018 in the United States,^3^ and this situation has developed into serious socioeconomic problems worldwide.

In Japan, the epidemic began to spread briefly around 1960, but it has rapidly declined due to amendments to the illicit drug control law and the drug banishment campaign in Japan. In 2016, the number of heroin arrests and the number of individuals arrested were three and zero, respectively, and the amount of heroin seized was low at 4.540 g in Japan.^4^ Although the indications for opioid use are increasing in Japan, its misuse or abuse has not yet become apparent. This has resulted in few opportunities to address and treat opioid use disorders in clinical psychiatric settings in Japan. However, in recent years, drug use while traveling abroad, trafficking by foreigners, and drug trafficking via the Internet has diversified the illicit sources of drugs. Given the increase in the number of foreigners, including tourists, the number of opioid abuses and heroin dependence in Japan may increase in the future.

The number of heroin addicts admitted at Tarumi Hospital in Kobe, Japan, began to increase around April 1998. Most of them were refugees from Southeast Asian descent. Historically, the first boat people came to Japan in May 1975, following which the first Vietnamese refugees arrived in Japan in 1978. Since the permission to settle in Kobe was granted, various problems encountered by Vietnamese living in Kobe were exposed by the Great Hanshin‐Awaji Earthquake in 1995, which led to the establishment of the nongovernmental organization “Vietnam in Kobe” in 2000. Since the first Vietnamese refugee heroin addict was admitted to the hospital in April 1998, the number has been increasing in hospital care.

Heroin addiction is characterized by painful symptoms of withdrawal. Although both the pleasure from drug use and the craving that comes from psychological dependence are strong enough, most of the sustained use of heroin abusers is mainly to avoid experiencing the pain of withdrawal symptoms. As to short‐acting heroin, a wide variety of autonomic symptoms, known as “autonomic storms,” emerge several hours after the last use of the substance. Autonomic symptoms, such as general malaise, slight fever and chills, sweating, goose bumps, tears, coughing, and yawning, begin to appear as well as severe pain, such as myalgia, arthralgia, and bone pain, followed by gastrointestinal symptoms such as diarrhea, nausea, and vomiting, accompanied by insomnia and intense anxiety and agitation. Commonly, most patients would visit the clinic to alleviate pain and to discontinue heroin. The treatment should begin with the discontinuation of heroin use (ie, detoxification from heroin). However, when our hospital started to accept heroin‐dependent patients, we experienced difficulties in the management of their withdrawal period. Patients were often secluded due to psychomotor agitation with distress of withdrawal symptoms, and we only administered fluids for gastrointestinal symptoms and anorexia. With regard to medication, sleeping pills, antipsychotics, and anxiolytics were mainly used as a symptomatic treatment for insomnia and anxiety caused by the pain of withdrawal symptoms, which were not much different from the treatment of psychotic disorders such as schizophrenia and other substance‐related disorders. In other words, medications were expected to improve insomnia, agitation, and restlessness associated with withdrawal symptoms, but they were largely ineffective in reducing the pain of withdrawal symptoms. To overcome this situation, buprenorphine (Lepetan®) treatment for opioid detoxification was started on a trial basis in 2002. Subsequently, buprenorphine replacement taper therapy (BRTT) was fully introduced in our hospital in 2005.^5^

Buprenorphine is a high‐potency partial agonist that acts on the μ‐opioid receptor. It is currently marketed in Japan as an injection and a suppository for postoperative pain, cancer pain, and myocardial infarction. In brief, buprenorphine is characterized by a high affinity for μ‐opioid receptors, a long blood half‐life, and low intrinsic activity.^6^ Consequently, its effect as an opioid is weaker than that of full agonists such as heroin. Due to the ceiling effect with buprenorphine,^7^ the effect is not further potentiated when the dose is increased beyond a certain level. In addition, even if a full agonist is administered during buprenorphine administration, other full agonists cannot replace buprenorphine due to the high affinity of the drug, preventing the onset of the effect of the full agonist. Furthermore, the duration of effectiveness in buprenorphine has an additional advantage because its dissociation rate from the μ‐opioid receptor is low. Because of these pharmacological characteristics, global standard guidelines such as the World Health Organization (WHO)^8^ and the UK's National Institute for Health and Care Excellence (NICE)^9^ recommend buprenorphine as a treatment option for the relief of withdrawal symptoms during detoxification. Although several other treatment options are also recommended according to these guidelines, narcotics must not be used to alleviate the symptoms of addiction or to treat the addiction of drug addicts according to domestic law in Japan. Therefore, we used buprenorphine, a non‐narcotic opioid commercially available in Japan, for the detoxification treatment of heroin. We briefly reported its efficacy and safety as an acceptable optional treatment for detoxification in patients with heroin dependence.^5^ However, because the number of cases has been limited and treatment opportunities for heroin addicts are rare nationwide, further study is required to validate the efficacy of BRTT.

In the present study, we examined the medical records of 106 heroin‐dependent patients who had been treated with buprenorphine to determine the effect of BRTT by comparing 26 patients who were admitted to the hospital without the administration of buprenorphine for the detoxification of heroin.

## 2 METHODS

### 2.1 Buprenorphine replacement and taper therapy

1. Establish an initial tentative daily dose of buprenorphine. We administered four ampules of 0.2 mg (0.8 mg) intramuscularly per day, as a start, to patients with standard heroin use. However, if we suspected that the patient could be highly tolerant based on the interview and treatment history, we administered six to eight ampules (1.2‐1.6 mg) per day. Conversely, three ampules (0.6 mg) injection of buprenorphine were initiated in cases with apparently low heroin use.
2. Many patients with heroin dependence usually use heroin until just before admission or until the morning after admission. Because of the persistent effects of heroin at the time of admission, we need to pay attention to the timing of administration, as buprenorphine may induce withdrawal symptoms. After admission, the patient should be monitored for some time while waiting for the onset of withdrawal symptoms. A tentative daily dose of buprenorphine will be administered starting from the time withdrawal symptoms are confirmed. At this point, the acute symptoms of buprenorphine intoxication should not be overlooked (bradycardia, hypotension, pupil constriction, respiratory depression, and delirium). Therefore, the drug should be administered in dose at intervals of 1‐3 hours and discontinued if signs of acute intoxication are observed while confirming safety. If the first day's dose is six ampules or more, two ampules should be administered at a time. Buprenorphine treatment was usually initiated in the late afternoon, but withdrawal symptoms are often insignificant at this point. In cases with high tolerance, withdrawal symptoms may become more severe late at night on the first day after admission, so additional doses may be administered as necessary while the patient is repeatedly examined to monitor the withdrawal symptoms.
3. On the morning of the second day in the hospital, withdrawal symptoms were assessed and the daily dose of buprenorphine was adjusted. If few withdrawal symptoms were observed, the dose of buprenorphine could be reduced from the day 1 buprenorphine dose and continued through day 4. If withdrawal symptoms were persistent but can be objectively and subjectively tolerated, the day 1 buprenorphine dose was maintained, administered in divided doses from morning to lights‐out, and that dose is continued through day 4. In cases where additional doses were administered on the first night or in case severe withdrawal symptoms emerged, such as restlessness, or patients heavily suffering from distress due to withdrawal symptoms, the dose of buprenorphine was increased from the day 1 buprenorphine dosage and additional doses were administered. While monitoring the withdrawal symptoms frequently, one to two ampules of buprenorphine (0.2‐0.4 mg) should be administered at 1‐ to 2‐hour intervals, with no upper limit for additional doses, until withdrawal symptoms improve. Withdrawal symptoms usually improve dramatically once the required dose is met. If additional doses are required, a higher dose is administered, and the dose is continued until day 4. In general, such a treatment course can largely eliminate withdrawal distress on the second day of hospitalization; then, withdrawal symptoms are significantly reduced by the third day (48‐72 hours later), which is regarded as the peak of withdrawal symptoms.
4. Starting on day 5, buprenorphine was gradually tapered off, one ampule every 2 days if the continuous dose by day 4 was low or one ampule per day if the continuous dose was high in the case with high tolerance. Withdrawal symptoms with buprenorphine may occur when buprenorphine is tapered at a faster pace. However, such withdrawal symptoms are much milder and less unbearable than those of heroin withdrawal. Taper off as slowly as possible is recommended, but people who are administered buprenorphine may seek a prompt reduction or eager to leave the hospital during the tapering off. The "endpoint (completion)" of BRTT was fixed as 2 days after the last administration of buprenorphine.

Withdrawal symptoms were assessed using the Clinical Opiate Withdrawal Scale (COWS).^10^ The instrument can be completed in approximately 2 minutes while talking with a patient and observing opioid withdrawal signs. It can be serially administered to track changes in the severity of opiate withdrawal symptoms over time or in response to treatment. This scale calculates the total number of withdrawal symptoms on a scale of 0 to 4 or 5 for each of the 11 withdrawal items. The score for each item reflects the severity of the sign or symptom, and the total scores were grouped as “mild (5‐12 points),” “moderate (13‐24),” “moderately severe (25‐36),” and “severe (more than 36).” We usually assessed the withdrawal symptoms at least three times a day during detoxification therapy in our hospital.

### 2.2 Participants

Based on the currently available medical records, we examined a total of 132 patients with heroin dependence who were admitted to our hospital for heroin detoxification between September 1, 1998, and July 31, 2020. To determine the efficacy of BRTT, those who had comorbid psychiatric disorders were excluded from the present study. Because this was a retrospective observational study based on medical records, informed consent was obtained from the subjects by means of opt‐out consent. Patient information was kept confidential and anonymous. The study and protocols were approved by the Ethics Committee of Tarumi Hospital and were in accordance with the Declaration of Helsinki.

The characteristics of the subjects—such as gender, age, nationality, and status of medical insurance—were obtained from their medical records. Factors such as length of hospital stay, duration of buprenorphine administration (days), maximum daily buprenorphine dose (mg), and total buprenorphine dose (mg) were extracted to evaluate the efficacy of BRTT. Furthermore, to assess the severity of withdrawal symptoms, the COWS score at the time of admission, the maximum of the withdrawal symptoms (maximum), and discharge were investigated for 85 subjects who could be confidently tracked through the medical records.

### 2.3 Data analyses

We used analysis of covariance (ANCOVA) to test the efficacy of BRTT on the length of hospital stay. The model used treatment group (BRTT vs non‐BRTT) as the main effect, with gender, age, history of hospitalization, nationality, and status of medical insurance as covariates. The non‐BRTT group comprised those who received any conventional treatment before the implementation of buprenorphine use. For the outcome measures, a log‐rank test was conducted to evaluate the completion (retention) rate in the treatment of detoxification between the two; “patient dropout” was considered when patients censored the detoxification treatment for their own reasons. In addition, the influences of potential confounding factors on the completion rate, buprenorphine dose (mg), and the COWS score were assessed using chi‐square test or one‐way analysis of variance, for each variable (gender, age, history of hospitalization, nationality, and status of medical insurance). Finally, Pearson's correlation analysis was performed to determine the relationship between the COWS and buprenorphine administration. Statistical analysis was performed using SPSS ver. 24.0 for Windows (SPSS Inc).

## 3 RESULTS

**FIGURE 1:**
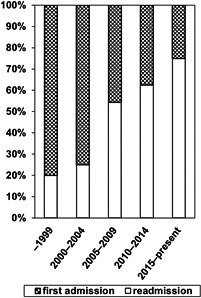
Annual admissions of heroin‐dependent patients in our hospital (1998‐2020 present). In each era, the number of readmissions (plaid) and first admission (solid) was expressed as percent (%)

**FIGURE 2:**
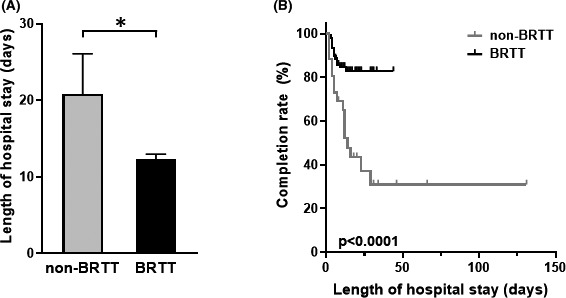
Comparison of hospitalization days (A) and treatment completion rate (B) before and after the introduction of buprenorphine replacement taper therapy. The length of hospital stay and completion rate for detoxification between BRTT (black) and non‐BRTT (gray) are presented in (A) and (B), respectively. *P < .001. BRTT, buprenorphine replacement therapy

The demographic characteristics of subjects with heroin dependence are shown in Table 1. Of the 132 eligible subjects, 106 subjects were heroin dependents who received BRTT and 26 subjects did not receive BRTT, as they had been hospitalized before the introduction of buprenorphine therapy for detoxification (non‐BRTT). As shown in Table 1, more than 90% of the patients were male and the age at admission was around 40 years old, both of these characteristics did not differ between the two groups. Of the 26 patients who received any conventional treatment (non‐BRTT), 20 were initially admitted and the remaining were readmitted. In contrast, of the 106 patients who received BRTT, 45 were initially admitted and 61 were readmitted. Although we previously reported that the number of hospitalizations with heroin dependence increased rapidly after the induction of BRTT, the present study demonstrated a gradual increase in the percentage of readmissions (Figure 1). The majority of subjects were of foreign nationality (mostly Southeast Asian nationality), and no significant changes in nationality were observed between the two groups. The length of hospital stay was significantly shorter in the BRTT group than in the non‐BRTT group (Table 1, Figure 2A: *P* = .0021). None of confounding factors affected the length of hospital stay. Furthermore, the completion rate of detoxification treatment was 84% in the BRTT group, which was significantly higher (Figure 2B: *P* < .0001) than in the non‐BRTT group (38%).

**TABLE 1:** Patients' characteristics

Treatment group	non‐BRTT		BRTT	
	N	ratio	N	Ratio
	Mean	%	Mean	%
Total number of subjects	26		106	
Readmission	6	23.1	61	57.5
Gender				
Male	24	92.3	100	94.3
Female	2	7.7	6	5.7
Age (y)	36.0		38.1	
(SD)	±10.1		±8.5	
Nationality				
Foreign country	22	84.6	97	84.9
Japan	4	15.4	9	15.1
Medical insurance				
Public insurance	19	73.1	72	67.9
Local public assistance	7	26.9	25	23.6
None	0	0	9	8.5
Length of hospital stay	20.8		12.3	

Abbreviation: BRTT, buprenorphine replacement therapy; SD, standard deviation.

**FIGURE 3:**
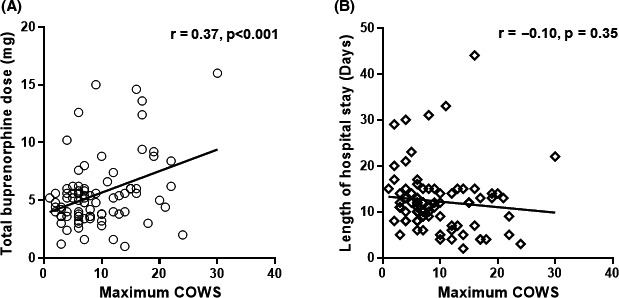
Correlations between the COWS score at maximum and total buprenorphine dose (A) and length of hospital stay (B) in patients with buprenorphine replacement taper therapy. COWS, Clinical Opiate Withdrawal Scale

The means of buprenorphine dosage, its dosing duration (days), and the COWS scores during detoxification with BRTT are summarized in Table 2. In the BRTT group, the maximum daily buprenorphine dose ranged depending on the subjects from the lowest dosage of 0.6 mg (three ampules) to the highest dosage of 6 mg (30 ampules). The total buprenorphine dose also varied widely from a minimal dosage of 1.6 mg (eight ampules) to a maximum dosage of 16 mg (80 ampules). The primary outcome measures, the COWS scores, revealed that withdrawal symptoms had clearly disappeared by the time of discharge (Table 2). Furthermore, the maximum scores of the COWS during detoxification were positively correlated with the total buprenorphine dose (Figure 3A: *r* = .37, *P* < .001), whereas there was no association between the maximum scores of COWS and the length of hospital stay (Figure 3B). Finally, potential confounding factors showed no significant effects on the completion rate, total buprenorphine dose (mg), and the maximum COWS scores (all *P* > .07).

**TABLE 2:** The summary of buprenorphine use and COWS scores in BRTT

	Bup use and COWS scores in BRTT
	Mean (SEM)
Duration of Bup administration (days)	8.2 (0.3)
Maximum daily Bup dose (mg)	1.1 (0.1)
Total Bup dose (mg)	5.3 (0.3)
COWS score	
Admission time	2.3 (0.2)
Maximum time	9.3 (0.7)
Discharge time	0.6 (0.2)

Abbreviations: BRTT, buprenorphine replacement taper therapy; Bup, buprenorphine; COWS, Clinical Opiate Withdrawal Scale; SEM, standard error.

## 4 DISCUSSION

To the best of our knowledge, the present study is the first report investigating the efficacy of BRTT in people with heroin dependence in Japan.

The length of hospital stay was significantly shorter for patients who received BRTT, and BRTT improved the completion rate of heroin detoxification, as compared to the non‐BRTT group. The current completion rate of treatment (84%) was consistent with previous reports^11^ ranging from 65% to 100% with buprenorphine. Given the changes in the COWS scores, the outcome measure for withdrawal symptoms, we believe that BRTT enables alleviation of withdrawal symptoms as a feasible detoxification treatment even in a Japanese clinical setting. The significant positive correlation between the maximum COWS scores and the total buprenorphine dose suggests that the total dosage of buprenorphine may consequently increase with the intensity of withdrawal symptoms during detoxification (eg, in cases with high tolerance). Nevertheless, the mild correlation between the two (*r* = 0.37) implies that buprenorphine dosage could be adjusted according to the situation by frequent interviews and observation for withdrawal symptoms, rather than by the COWS scores alone in clinical practice. There was no correlation between the maximum COWS scores and length of hospital stay, suggesting that stronger symptoms during the withdrawal phase did not necessarily lead to prolonged hospitalization. Instead, BRTT may have contributed to a shorter hospital stay for heroin addicts requiring detoxification.

Detoxification with buprenorphine use is completely different from conventional treatments such as immediate abstinence from heroin. Indeed, the introduction of BRTT had a significant impact on those who treated patients with heroin dependence in our hospital. The medical staff in psychiatric hospitals tend to feel distressed when accepting people who exhibit hostility and aggressive behavior, physical problems, or uncertain outlooks about inpatient care. After BRTT was introduced in our hospital, patients with heroin dependence rarely became agitated due to withdrawal symptoms. Although mild side effects such as constipation and delirium were observed, we could have a better outlook on the process of withdrawal symptoms and can prepare to deal with exacerbations. In addition, linguistic communication was a major problem because the majority of patients were non‐Japanese. However, the introduction of buprenorphine can contribute to a significant reduction in anxiety and distress for medical staff.

We found an increase in readmission rates following the introduction of detoxification therapy with buprenorphine. One possible explanation for the higher rates of readmission is that treatment with buprenorphine greatly alleviated the distress of withdrawal symptoms, which may have resulted in patients who were eager to enter the hospital again for detoxification in the case of a relapse without hesitation. Importantly, higher rates of readmission indicated the importance of maintenance therapy. Indeed, treatment for substance abuse disorder should not only be restricted to short‐term detoxification and symptomatic treatment, but also include maintaining long‐term follow‐up with psychosocial approaches such as cognitive behavioral therapy, contingency management, and utilizing mutual aid groups such as narcotics anonymous and other social supports. The goal is to prevent high‐risk behaviors that lead to relapse and promote social adjustment and human maturity. In other words, detoxification should be only the beginning of treatment for people with heroin dependence. In fact, we found that only a few patients attended the hospital after they were discharged in our hospital. Although the introduction of buprenorphine increased the motivation for detoxification treatment, it was extremely difficult to translate into the treatment for long‐term maintenance of abstinence under the current conditions, at least in our Japanese hospital. For this reason, maintenance therapy with opioid receptor full agonists or opioid receptor partial agonists may be desirable in addition to psychosocial interventions when considering the WHO guidelines.^8^ For example, the use of the long‐acting full opioid agonist methadone began in the United States in the 1960s. Similarly, the opioid receptor partial agonist buprenorphine is used for several advantages such as safety and efficacy. Therefore, its use for maintenance therapy is currently recommended as a standard pharmacotherapy worldwide.^12^ In contrast, the treatment strategy for opioid use disorder has not been modified or updated in the last half century because Japan, fortunately, did not suffer from the wave of the opioid crisis. As previously stated, Japanese law prohibits maintenance therapy using methadone designated as a narcotic, and low‐dose injections and suppositories of buprenorphine, which is available in Japan, can be used for inpatient detoxification therapy but not for maintenance therapy required for long‐term care. There have been increasing concerns about the diversion and misuse of buprenorphine or methadone pharmacotherapy.^13^ However, considering that many countries offer the option of replacement, such as tapered off and maintenance therapy with methadone and buprenorphine, treatment options for opioid dependence might become outdated in Japan.

Based on a large number of past practices, we demonstrated the efficacy of BRTT in the detoxification of heroin dependence. However, the present study has some potential limitations. First, the BRTT was exclusively performed in our hospital, and the majority of the patients were foreign nationals, all of which may have caused selection bias. Second, all subjects received psychotropic medications at least once during hospitalization, and people who had comorbid other psychiatric disorders were excluded from this study. In addition, factors affecting BRTT treatment, such as heroin dosage, duration of heroin use, developmental history, and socioeconomic factors, were not considered in the present study. Prospective studies should validate the effectiveness of BRTT while considering the extent to which these factors can affect BRTT.

Finally, detoxification with buprenorphine is the only medication that can be administered to people with heroin dependence in Japan. Psychosocial interventions play crucial roles in heroin addicts, who are typically chronic and relapsing. However, we sincerely hope that the Japanese law that prohibits maintenance therapy using methadone will be reviewed in the future and that high‐dose buprenorphine sublingual tablets will be introduced as a treatment option for heroin dependence in Japan.

## CONFLICT OF INTEREST

The authors have no conflicts of interest to declare.

## AUTHOR CONTRIBUTIONS

TN, TA, TK, YY, and KO were involved in data collection. TN and S. K. analyzed the data and wrote the manuscript. NY and To.K. supervised the entire project. All authors have read and approved the final manuscript.

## APPROVAL OF THE RESEARCH PROTOCOL BY AN INSTITUTIONAL REVIEWER BOARD

This study was approved by the Ethics Committee of Tarumi Hospital, Japan.

## INFORMED CONSENT

Informed consent was obtained from the subjects by means of opt‐out consent.

## Supporting information

Table S1

## Data Availability

We cannot deposit the raw demographic information of each participant in a public repository, since we did not obtain informed consent of the participants to make it publicly available. However, these data required for the analyses are available from the corresponding author on reasonable request. For Figures 2 and 3, the data that support the findings of this study are available in the supplementary material of this article.

## References

[1] Volkow ND , McLellan AT . Opioid abuse in chronic pain—misconceptions and mitigation strategies. N Engl J Med. 2016;374:1253–63. 27028915 10.1056/NEJMra1507771

[2] Madras BK . The president's commission on combating drug addiction and the opioid crisis: origins and recommendations. Clin Pharmacol Ther. 2018;103:943–5. 29570781 10.1002/cpt.1050

[3] Han B , Volkow ND , Compton WM , McCance‐Katz EF . Reported heroin use, use disorder, and injection among adults in the United States, 2002–2018. JAMA. 2020;323:568–71. 32044936 10.1001/jama.2019.20844 PMC7042840

[4] Ministry of Health, Labour and Welfare . [Cited 2020 Sep 2]. Available from https://www.mhlw.go.jp/bunya/iyakuhin/yakubuturanyou/gyousei-gaikyo/torishimari.html. (in Japanese)

[5] Katsuro A . Experience of using injectable formulation of buprenorphine for the detoxification treatment of heroin dependence patients. Jpn J Alcohol Drug Depend. 2009;44:155–6. (in Japanese). 19618840

[6] Orman JS , Keating GM . Buprenorphine/naloxone: a review of its use in the treatment of opioid dependence. Drugs. 2009;69:577–607. 19368419 10.2165/00003495-200969050-00006

[7] Walsh SL , Preston KL , Stitzer ML , Cone EJ , Bigelow GE . Clinical pharmacology of buprenorphine: ceiling effects at high doses. Clin Pharmacol Ther. 1994;55:569–80. 8181201 10.1038/clpt.1994.71

[8] WHO Guidelines Approved by the Guidelines Review Committee , , Guidelines for the psychosocially assisted pharmacological treatment of opioid dependence. Geneva: World Health Organization; 2009. 23762965

[9] National Collaborating Centre for Mental Health . NICE clinical guidelines, No. 52. Drug misuse: opioid detoxification. Leicester: British Psychological Society, 2008. 21452460

[10] Wesson DR , Ling W . The Clinical Opiate Withdrawal Scale (COWS). J Psychoactive Drugs. 2003;35:253–9. 12924748 10.1080/02791072.2003.10400007

[11] Gowing L , Ali R , White J . Buprenorphine for the management of opioid withdrawal. Cochrane Database Syst Rev. 2002:Cd002025. 12076434 10.1002/14651858.CD002025

[12] Kleber HD . Pharmacologic treatments for opioid dependence: detoxification and maintenance options. Dialogues Clin Neurosci. 2007;9:455–70. 18286804 10.31887/DCNS.2007.9.2/hkleber PMC3202507

[13] Lofwall MR , Walsh SL . A review of buprenorphine diversion and misuse: the current evidence base and experiences from around the world. J Addict Med. 2014;8:315–26. 25221984 10.1097/ADM.0000000000000045 PMC4177012

